# Efficacy of Sequential Hepatic Vein Embolization Following Portal Vein Embolization in Promoting Regeneration of Liver Volume and Function Before Right‐Sided Major Hepatectomy

**DOI:** 10.1002/ags3.70085

**Published:** 2025-08-27

**Authors:** Thanh Tung Lai, Kosuke Matsui, Hideyuki Matsushima, Hidekazu Yamamoto, Gozo Kiguchi, Hisashi Kosaka, Van Khanh Nguyen, Yasuhiro Ueno, Shuji Kariya, Masaki Kaibori

**Affiliations:** ^1^ Department of Hepatobiliary Surgery Kansai Medical University Osaka Japan; ^2^ Department of Surgery Hanoi Medical University Hanoi Vietnam; ^3^ Internal Gastroenterology Department VNU University of Medicine and Pharmacy Hanoi Vietnam; ^4^ Department of Radiology Kansai Medical University Osaka Japan

**Keywords:** embolization, hepatectomy, hepatic vein, liver regeneration, portal vein

## Abstract

**Background/Purpose:**

This study compared the efficacy of sequential hepatic vein embolization (HVE) following portal vein embolization (PVE) with PVE alone in promoting both the volume and function of the future remnant liver (FRL) before right‐sided major hepatectomy.

**Methods:**

All patients underwent preoperative PVE with or without sequential HVE, followed by right hepatectomy ± extended from 2018 to 2023. Changes in FRL volume and function were analyzed and compared between groups, with liver function assessed using technetium‐99m diethylenetriaminepentaacetic acid‐galactosyl‐human serum albumin (^99m^Tc‐GSA) scintigraphy.

**Results:**

Eight patients underwent sequential PVE–HVE, while 24 underwent PVE alone. All patients underwent embolization without severe complications. The median regeneration rate of FRL volume was significantly higher in the sequential HVE–HVE group at 35.5% (20.6%–54.0%) compared with 26.2% (19.2%–31.0%) in the PVE group (*p* = 0.017). Moreover, the median FRL function regeneration rate was 59.5% (41.6%–80.9%) in the sequential PVE–HVE group, markedly greater than the rate of 25.2% (1.2%–41.4%) in the PVE group (*p* = 0.014). There was no significant difference in surgical outcomes between groups.

**Conclusions:**

Sequential PVE–HVE is safe and practical, leading to a significant increase in both the volume and function of the FRL.

## Graphical abstract

Sequential hepatic vein embolization (HVE) following portal vein embolization (PVE) significantly improves both the volume and function of the future remnant liver (FRL) compared to PVE alone. In this study, the FRL regeneration rates for volume and function were markedly higher and faster in the PVE–HVE group, with no differences in surgical outcomes or severe complications. Sequential PVE–HVE is a safe and effective approach for enhancing liver regeneration before major right‐sided hepatectomy.

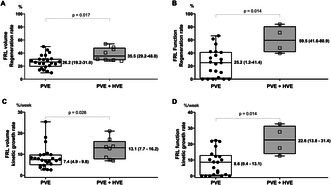

## 1 Introduction

Liver resection stands as the most radical treatment for various types of liver cancer, such as hepatocellular carcinoma (HCC), intrahepatic cholangiocarcinoma (ICC), perihilar cholangiocarcinoma (PHCC), and colorectal liver metastases (CRLM) [1]. However, the indications for liver resection are limited by several factors, such as extensive tumor invasion, liver fibrosis, and multifocal bilobar tumor. Regardless of the cause, the primary concerns revolve around limited remnant liver function. This serves as a barrier to recommending liver resection, because the foremost priority is to ensure adequate liver function after hepatectomy to reduce the risk of liver failure.

One way to extend the indications for liver resection in such cases is through portal vein embolization (PVE), which was introduced in the late 1980s in Japan and was first reported by Kinoshita in 1986. Currently, this technique is being employed in most liver surgery centers in many countries as a standard procedure to increase liver volume before major hepatectomy. However, PVE does not always promote the regeneration of the remnant liver effectively. Approximately 4%–34% of patients are unable to undergo hepatectomy post‐embolization [2, 3]. Several reasons account for this, such as the drifting of the embolization material, which leads to recanalization of the portal vein (PV), alterations in PV anatomy, liver fibrosis impairing the regenerative capacity, compensatory hepatic arterial hyperperfusion, remaining peripheral PV branches owing to arterioportal shunts or too‐proximal PV embolization [3, 4, 5]. Some factors can be mitigated by carefully evaluating multiple aspects in each case and adjusting the PVE technique accordingly. However, certain factors cannot be improved solely by using PVE. Moreover, the waiting time for liver hypertrophy is quite lengthy (about 4–8 weeks), during which disease progression may occur, particularly in cases of highly aggressive or large tumors, making liver resection unfeasible for these patients [3, 4, 5].

Another method to increase liver volume, known as combined PVE and hepatic vein embolization (HVE), has emerged in recent years. This approach, first reported by Nagino et al. in 2003 [6], is believed to be more effective than PVE alone, and various combinations of PVE and HVE techniques have been performed in several centers worldwide. These procedures have shown superior effectiveness in increasing liver volume compared to PVE alone. Examples include salvage HVE after PVE [7], sequential HVE after PVE [8], radiological simultaneous portal hepatic vein embolization (RASPE), liver venous deprivation (LVD), biembolization (BE) [9, 10, 11, 12, 13, 14], and extended liver venous deprivation (eLVD) [15].

The main aim of this technique is to improve the function of the remaining liver. However, most studies comparing the effectiveness of combined PVE and HVE versus PVE alone assess liver function through blood tests and future remnant liver volume (FRLV) [9, 10, 11]. Technetium‐99m diethylenetriamine pentaacetic acid galactosyl human serum albumin (^99m^Tc‐GSA) scintigraphy is a handy tool for evaluating the function of each liver segment [16].

The goal of the present study was to compare the changes in volume and function of the future remnant liver (FRL), assessed using ^99m^Tc‐GSA scintigraphy, between sequential HVE following PVE versus PVE alone before right‐sided major hepatectomy.

## 2 Materials and Methods

### 2.1 Study Design

This was a retrospective analysis of data from patients treated at Kansai Medical University Hospital between March 2018 and June 2023, with all patients scheduled for major hepatectomy regardless of diagnosis with initial limited FRL indicated for PVE with or without HVE to induce liver hypertrophy. The indication for FRL hypertrophy before major hepatectomy using PVE or sequential PVE–HVE in our center was a FRLV/total liver volume (TLV) ratio < 25%–30% in individuals with a normal liver or < 35%–40% in cases with underlying liver disease (cirrhosis, chronic hepatitis) [17]. All of our patients undergo ^99m^Tc‐GSA scintigraphy for the evaluation of liver function before surgery, which allows for a thorough assessment of each liver section's functionality. PVE or sequential PVE–HVE was also indicated when the maximal removal rate of ^99m^Tc‐GSA (GSA‐Rmax) of the FRL was < 0.15 mg/min per 50 kg of body weight (BW) [16]. This study included patients who received either PVE or PVE followed by HVE and subsequently underwent right‐sided major hepatectomy after embolization. Other patients were excluded from the study. This study was approved by the ethics committee of Kansai Medical University (reference number: KMU 2024102). The primary outcome was to compare the extent of the volume and function of the FRL following PVE with or without HVE before right‐sided major hepatectomy. A secondary outcome was to compare surgical outcomes and complications between groups.

### 2.2 Embolization Procedure

PVE and HVE techniques have been described in detail previously [8]. Ultrasound was performed prior to the embolization to identify the access route to the right PV. Intravenous anesthesia combined with local anesthesia was used. PVE was performed under ultrasound and fluoroscopic guidance. A 10‐cm, 22‐gauge needle 10 cm was used to puncture a right portal branch. A 0.016‐in., 160‐cm IRIS guide wire (Asahi Intecc, Aichi, Japan) was advanced through the needle for access. It was then replaced with a 150‐cm, 0.035‐in. RADIOFOCUS guide wire (Temuro, Tokyo, Japan). A 4‐Fr, 11‐cm sheath was first inserted and subsequently replaced with a 5‐Fr, 11‐cm sheath over the guide wire. Portal venography and CT portography were performed using a 5‐Fr, 65‐cm pigtail catheter. Embolization was performed using a mixture of *n*‐butyl cyanoacrylate (NCBA) and lipiodol with a ratio of 1:8 for right portal branches with or without segment 4 PV branches, and a ratio of 1:2 for tract embolization via microcatheters of 2.2‐Fr, 110 cm (SIRABE; Medikit, Tokyo, Japan) or 2.7‐Fr, 110 cm (Sniper; Temuro, Tokyo, Japan). Fluoroscopic monitoring was performed every 5 min for 20 min to assess the mixture of NBCA: lipiodol migration. Finally, computed tomography (CT) confirmed technical success and the absence of bleeding or other complications.

HVE was performed after PVE proactively, a few days later, through the right transjugular or transfemoral approach. Initially, a 4‐Fr sheath was inserted, subsequently replaced with an 8‐Fr sheath, and hepatic venography was performed. Target veins included the right hepatic vein branches and the inferior right hepatic vein, if present. Based on pre‐procedural CT and hepatic venography, selected branches were embolized using an Amplatzer vascular plug (AVP) II and/or 4 combined Interlock‐35 Coils or EMBOLD Fibered Coils. Post‐embolization angiography confirmed complete occlusion of the treated veins without central flow.

### 2.3 Preoperative Variables

All data were taken from the hospital's electronic data storage system. Blood test results were collected around 1 week before surgery. The albumin–bilirubin (ALBI) score was calculated using only serum albumin and total bilirubin [(log10 bilirubin [micromol/L] × 0.66) + albumin [g/L] × −0.085]. The time between embolization and imaging or surgery was calculated from the day PVE was performed to the day of imaging assessment before surgery or the day of surgery, respectively. Computed tomography (CT) was performed on all patients before and after embolization. The liver function of all patients was assessed by ^99m^Tc‐GSA scintigraphy before surgery.

The regeneration rate of FRLV (%) was determined using the formula: [(FRLV (mL) before surgery − FRLV (mL) before embolization) × 100]/FRLV (mL) before embolization. The kinetic growth rate (KGR) of FRLV represents the degree of hypertrophy at the initial volume assessment divided by the time elapsed since embolization, which was calculated as follows: FRLV regeneration rate (%)/time between embolization and imaging (weeks) with the unit of %/week.

Liver function evaluated by ^99m^Tc‐GSA scintigraphy was expressed through the region's maximal removal rate (Rmax) of ^99m^Tc‐GSA (mg/min) index. The regeneration rate of future remnant liver function (FRLF) of each case was calculated as follows: [(FRLF (mg/min) before surgery − FRLF (mg/min) before embolization) × 100]/FRLF (mg/min) before embolization. Similarly, the KGR of FRLF describes the functional hypertrophy rate per unit time since embolization, which was calculated as follows: FRLF regeneration rate (%)/time between embolization and imaging (weeks) with the unit of %/week.

### 2.4 Surgery

Surgery was performed by expert hepatobiliary surgeons. Major hepatectomy was defined as the resection of four or more liver segments [18]. Patients included in this study underwent a standard right hepatectomy (segments 5–8) only or combined with other surgery, such as segment 1 resection and/or segment 4 resection and/or partial resection of the left liver and/or extrahepatic bile duct resection (EHBDR). Surgical procedures were classified using the “New World” terminology for hepatectomy [19]. Data were recorded during hospitalization, and morbidity and mortality were assessed 90 days postoperative. The surgical margin was categorized as either positive or negative. A positive margin was defined as the presence of tumor cells at the line of transection, while a negative margin demonstrated their absence, both determined by histologic examination. Postoperative complications were reported based on the classification proposed by Dindo and Clavien [20]; significant complications had a grade ≥ III. Posthepatectomy liver failure (PHLF) was defined according to the International Study Group of Liver Surgery (ISGLS) [21]. Postoperative mortality was defined as death from any cause within 90 days of surgery.

### 2.5 Data Source and Management

All data were collected and analyzed at the Department of Hepatobiliary Surgery of Kansai Medical University Hospital. Demographic data were obtained retrospectively from maintained databases and electronic medical records. Liver volume was calculated by experienced hepatobiliary surgeons before surgery utilizing the 3D image analysis system “SYNAPSE VINCENT” by Fujifilm Co. Ltd., Tokyo, Japan. Liver function was evaluated by radiologists using ^99m^Tc‐GSA scintigraphy before surgery with a cutting line in single‐photon emission computed tomography (SPECT) with the CT scan for each case that was suggested by hepatobiliary surgeons before surgery.

### 2.6 Statistical Methods

Data are expressed as numbers with percentages or medians and ranges from the 25th to the 75th percentile. Fisher's exact test was used for nominal scale data. The Mann–Whitney *U* test was performed for continuous variables. Paired variables were compared using the Wilcoxon signed rank test. Differences were considered statistically significant at *p* < 0.05. All statistical analyses were performed with IBM SPSS statistic version 25.

## 3 Results

### 3.1 Patient Selection and Background Characteristics

**FIGURE 1:**
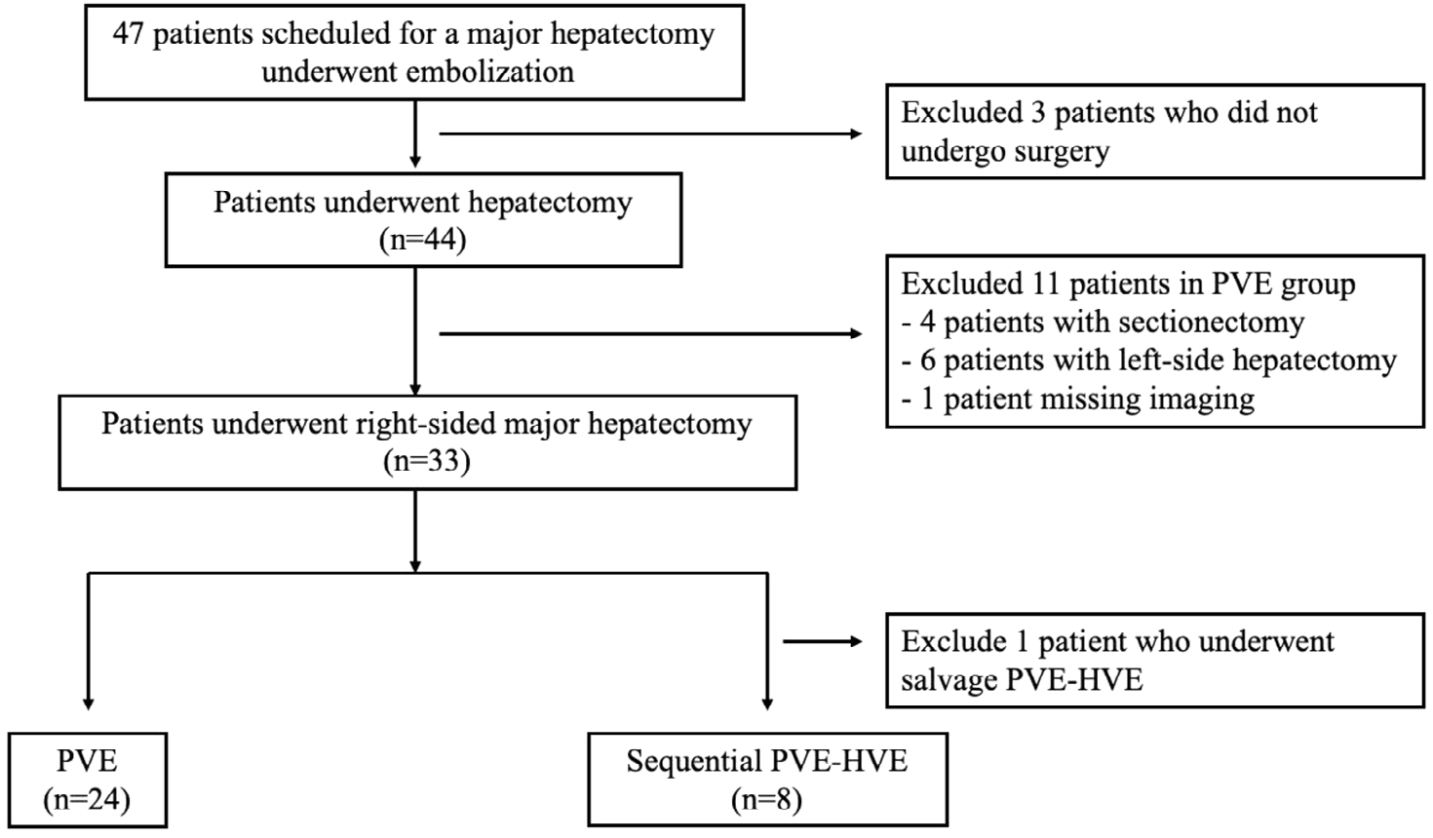
Study flowchart.

Between March 2018 and June 2023, 47 patients scheduled for major hepatectomy with limited FRLV underwent PVE with or without HVE after PVE. From March 2018 to July 2022, PVE alone was performed, while from August 2022 to June 2023, sequential PVE–HVE was used for all patients. Among 47 patients, 15 were excluded because of refusal of surgery (PVE, *n* = 1), discontinuation of surgery for tumor progression (PVE, *n* = 2; sequential PVE–HVE, *n* = 1), left‐side hepatectomy (PVE, *n* = 6), minor hepatectomy (PVE, *n* = 3), and missing imaging before embolization (PVE, *n* = 1); one patient underwent HVE after insufficient PVE, in which HVE was performed 31 days after PVE as salvage therapy. Briefly, 24 patients underwent PVE alone, and eight patients underwent sequential PVE–HVE; those who received right‐sided major hepatectomy after the embolization were included in this study. Patient selection is shown in Figure 1.

Patients underwent hepatectomy for CRLM (PVE, *n* = 12; sequential PVE–HVE, *n* = 1), ICC (PVE, *n* = 4; sequential PVE–HVE, *n* = 2), HCC (PVE, *n* = 5; sequential PVE–HVE, *n* = 2), PHCC (PVE, *n* = 3; sequential PVE–HVE, *n* = 2), gallbladder cancer (sequential PVE–HVE, *n* = 1), and intraductal papillary neoplasm of the bile duct (sequential PVE–HVE, *n* = 1).

There was no significant difference between groups in terms of preoperative patient characteristics, which are summarized in Table 1.

**TABLE 1:** Background characteristics.

Variable	PVE (*n* = 24)	Sequential PVE–HVE (*n* = 8)	*p*
Age, year	72.5 (69–77)	72.0 (68–81)	0.861
Gender, male	16 (66.7)	5 (62.5)	1.000
Hepatitis, HBV/HCV/NBNC	2/1/21	0/0/8	1.000
Body mass index, kg/m^2^	22.6 (20.2–25.7)	23.7 (20.7–25.0)	0.965
WBC, 10^9^/L	5.9 (4.7–7.1)	5.3 (4.6–5.7)	0.257
Hemoglobin, g/L	12.2 (10.3–13.0)	10.8 (9.3–13.0)	0.327
CRP, mg/L	0.35 (0.24–0.94)	0.80 (0.51–1.64)	0.101
Preoperative liver function			
AST, UI/L	33 (25–39)	29 (24–60)	0.896
ALT, UI/L	24 (17–28)	25 (12–36)	0.948
PLT, G/L	22.5 (15.8–27.8)	21.4 (15.0–26.8)	0.695
Prothrombin, %	94.3 (90.1–99.2)	97.3 (88.1–111.7)	0.396
Total bilirubin, mg/dL	0.6 (0.4–0.7)	0.7 (0.5–1.0)	0.086
Albumin, mg/dL	3.6 (3.1–3.9)	3.6 (3.2–3.7)	0.458
ICG R15, %	14.6 (8.9–19.4)	14.0 (8.7–18.9)	0.794
ALBI score	−2.44 (−2.61 to −2.01)	−2.25 (−2.42 to −1.99)	0.296
Child–Pugh score, 5:6:7	15:6:3	5:3:0	0.708
Primary disease			0.288
Colorectal liver metastases	11 (45.8%)	1 (12.5%)	
Intrahepatic cholangiocarcinoma	4 (16.7%)	2 (25%)	
Hepatocellular carcinoma	5 (20.8%)	2 (25%)	
Perihilar cholangiocarcinoma	3 (12.5%)	2 (25%)	
Gallbladder cancer	0 (0%)	1 (12%)	
Intraductal papillary neoplasm of bile duct	1 (4.2%)	0 (0%)	

*Note:* Data are shown as median (25th percentile to 75th percentile) or *n* (%).

Abbreviations: ALBI score, albumin–bilirubin score; ALT, alanine aminotransferase; AST, aspartate aminotransferase; CRP, C‐reactive protein; HBV, hepatitis B virus; HCV, hepatitis C virus; HVE, hepatic vein embolization; ICG‐R15, indocyanin retention rate after 15 min; NBNC, non‐B, non‐C; PLT, platelet count; PVE, portal vein embolization; WBC, white blood cell.

### 3.2 Radiological Outcomes

Among eight patients in the sequential PVE–HVE group, following PVE, six underwent right hepatic vein (RHV) embolization only, and two underwent both RHV and IRHV embolization; seven patients received the right transjugular approach, and one patient received the right transfemoral approach. No severe complications were observed during or after PVE and HVE that required reintervention or surgery. Two patients in the PVE group experienced intra‐abdominal bleeding, detected by follow‐up CT immediately after the procedure due to a small amount of fluid collection around the liver; however, no active bleeding was observed, and no additional intervention or blood transfusion was required. The minor complications are summarized in Table S1. The median time interval from PVE to HVE was 5 (3–7) days.

**FIGURE 2:**
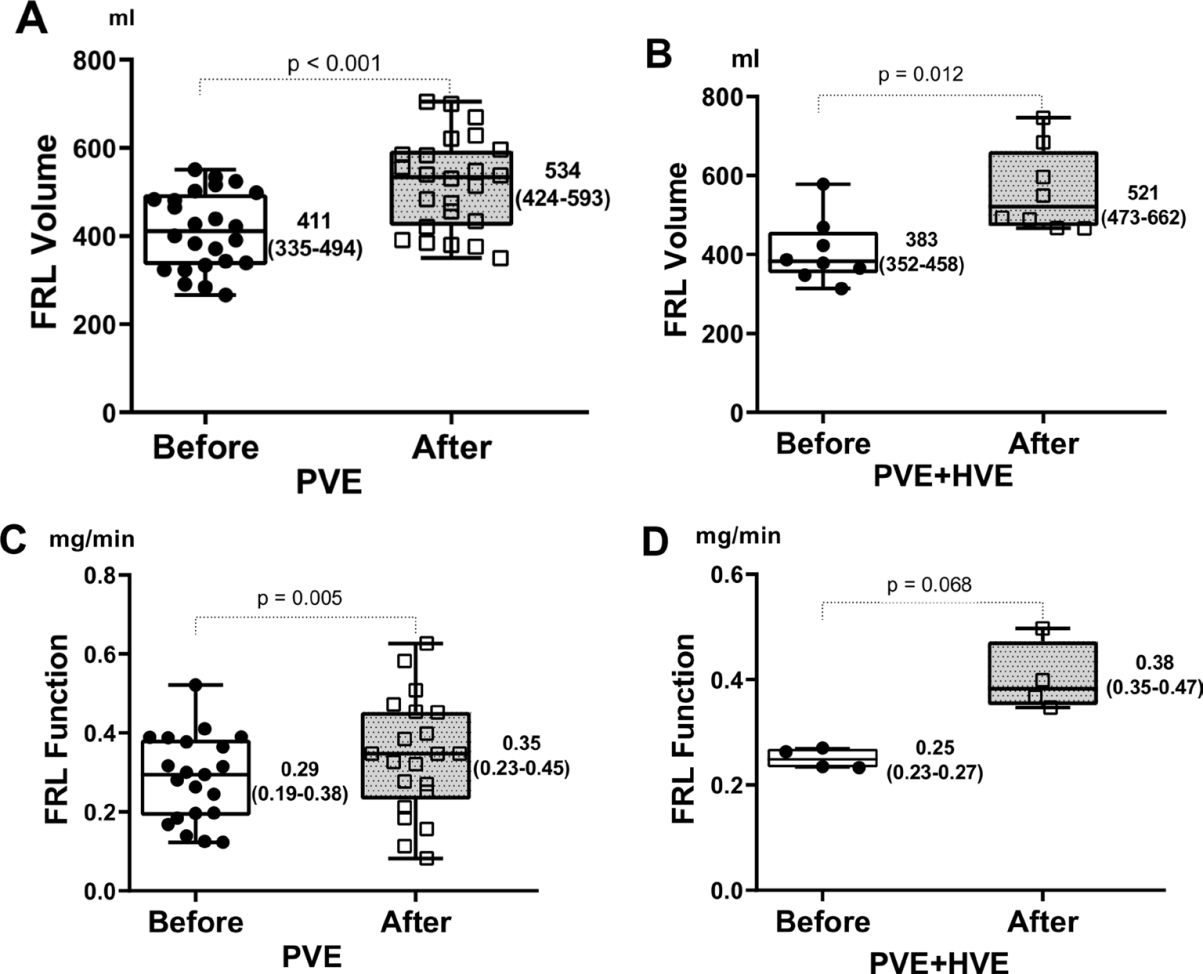
Comparative increase in FRL volume and function following embolization. (A) FRL volume before and after PVE (*n* = 24). (B) FRL volume before and after sequential PVE–HVE (*n* = 8). (C) FRL function before and after PVE (*n* = 21). (D) FRL function before and after sequential PVE–HVE (*n* = 4). FRL, future remnant liver; HVE, hepatic vein embolization; PVE, portal vein embolization. FRL volume was measured by computed tomography using Synap Vincent software (mL), and FRL function was measured by using ^99m^Tc‐GSA scintigraphy (mg/min). The horizontal bar inside the boxes indicates the median, and the lower and upper ends of the boxes represent the first and the third quartiles. The whiskers indicate the minimum and maximum values. All individual data points are displayed.

Figure 2 illustrates the changes in FRLV and FRLF before and after embolization in each group. FRLV increased significantly following embolization in both groups, as shown in Figure 2A,B. All patients in this study were assessed for liver function after embolization by ^99m^Tc‐GSA scintigraphy. Among 32 patients, 25 were evaluated by scintigraphy both before and after embolization (PVE, *n* = 21 and sequential PVE–HVE, *n* = 4). After embolization, FRLF increased significantly in the PVE group (Figure 2C), whereas no statistically significant difference was observed in the sequential PVE–HVE group (Figure 2D). The changes in TLV, FRLV/TLV ratio, FRLV/BW ratio, and total liver function after embolization in both groups are presented in detail in Table S2.

**FIGURE 3:**
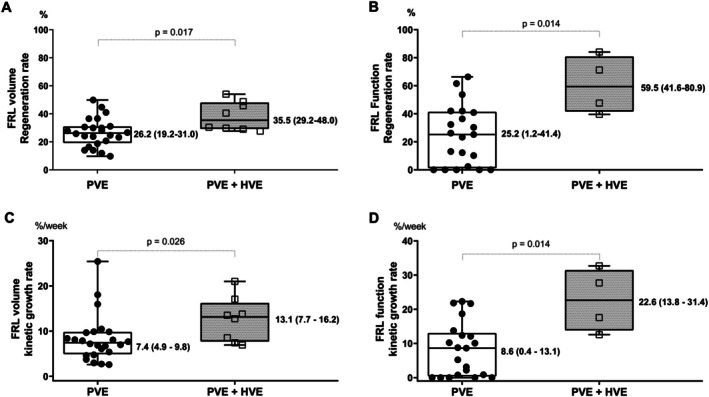
Comparative regeneration rate and KGR in FRL volume and function after embolization between PVE and sequential PVE–HVE groups. (A) FRL volume regeneration rate in the PVE group (*n* = 24) and sequential PVE–HVE group (*n* = 8). (B) FRL function regeneration rate in the PVE group (*n* = 21) and sequential PVE–HVE group (*n* = 4). (C) FRL volume KGR in the PVE group (*n* = 24) and sequential PVE–HVE group (*n* = 8). (D) FRL function KGR in the PVE group (*n* = 21) and sequential PVE–HVE group (*n* = 4). FRL, future remnant liver; HVE, hepatic vein embolization; KGR, kinetic growth rate; PVE, portal vein embolization. The horizontal bar inside the boxes indicates the median, and the lower and upper ends of the boxes represent the first and the third quartiles. The whiskers indicate the minimum and maximum values. All individual data points are displayed.

A comparison of the regeneration rates and KGR of FRLV and FRLF between groups is shown in Figure 3. The median regeneration rate of FRLV was 26.2% (19.2%–31.0%) in the PVE group, significantly lower than the 35.5% (29.5%—48.0%) observed in the sequential PVE–HVE group (*p* = 0.017, Figure 3A). Moreover, the median regeneration rate of FRLF was 25.2% (1.2%–41.4%) in the PVE group, compared with a significantly higher rate of 59.5% (41.6%–80.9%) in the sequential PVE–HVE group (*p* = 0.014, Figure 3B).

The KGR results were consistent with the regeneration rate findings. The median KGR of FRLV and FRLF in the PVE group was 7.4 (4.9–9.8) and 8.6 (0.4–13.1) %/week, which was significantly lower than the rates in the sequential PVE–HVE group, recorded at 13.1 (7.7–16.2) and 22.6 (13.8–31.4) %/week (Figure 3C,D, *p* = 0.026 and *p* = 0.014), respectively.

### 3.3 Surgical Outcomes

The median waiting time from PVE to surgery was 30.8 (26.2–39.1) days in the PVE group and 27.5 (26.1–34.9) days in the sequential PVE–HVE group (*p* = 0.317).

Surgical outcomes are shown in Table 2. One patient in the PVE group with a background of chronic heart failure, previous coronary artery bypass grafting, and pacemaker implantation died on postoperative Day 87 from renal and hepatic failure. The median operative time, blood loss during surgery, and rate of occurrence of major complications were not significantly different between the two groups.

**TABLE 2:** Surgical outcomes.

Variable	PVE (*n* = 24)	Sequential PVE–HVE (*n* = 8)	*p*
Surgical Procedure			0.209
H5678	13 (54.2%)	3 (37.5%)	
H56784′	1 (4.2%)	1 (0%)	
H5678‐B	1 (4.2%)	0 (0%)	
H145678	3 (12.5%)	0 (0%)	
H145678‐B	2 (8.3%)	0 (0%)	
H15678	2 (8.3%)	0 (0%)	
H15678‐B	2 (8.3%)	3 (37.5%)	
H15678/2′3′	0 (0%)	1 (12.5%)	
Operative time, min	442 (335–557)	405 (378–599)	0.473
Blood loss, mL	1216 (616–1702)	962 (918–1770)	0.965
Intraoperative blood transfusion, yes	10 (41.7%)	6 (75.0%)	0.220
Surgical margins			1.000
Negative	20 (83.3%)	7 (87.5%)	
Positive	4 (16.7%)	1 (12.5%)	
Blood test POD 5			
Total bilirubin, mg/dL	1.4 (1.0–2.8)	1.7 (1.2–3.3)	0.601
Prothrombine time, %	66.6 (54.5–77.2)	68.7 (62.7–89.1)	0.459
Prothrombine time—INR	1.23 (1.14–1.35)	1.21 (1.07–1.27)	0.433
Postoperative complications, *n* (%)			
Intraabdominal abscess	4 (16.7%)	0 (0%)	0.550
Ascites	15 (62.5%)	4 (50%)	0.684
Biliary leak	3 (12.5%)	0 (0%)	0.555
Acute cholangitis	7 (29.2%)	4 (50%)	0.397
Pneumothorax	1 (4.2%)	0 (0%)	1.000
Clavien–Dindo classification, ≥ IIIa			1.000
Yes	10 (41.7%)	2 (25.0%)	
No	14 (58.3%)	6 (75.0%)	
Posthepatectomy liver failure			0.443
No	10 (41.7%)	2 (25%)	
Grade A	7 (29.2%)	2 (25%)	
Grade B	5 (20.8%)	4 (50%)	
Grade C	2 (8.3%)	0 (0%)	
90‐day post‐operative mortality, yes	1 (4.2%)	0 (0%)	1.000

*Note:* Data are shown as median (25th percentile to 75th percentile) or *n* (%). Posthepatectomy liver failure was based on the international study group of liver surgery (ISGLS) classification. Surgical procedures were classified according to the “New World” Terminology for Hepatectomy.

Abbreviations: HVE, hepatic vein embolization; INR, international normalized ratio; POD, postoperative day; PVE, portal vein embolization.

## 4 Discussion

This study determined that sequential PVE–HVE is a safe procedure that improves the efficacy of liver regeneration before major hepatectomy compared to PVE alone in terms of both volume and function of FRL using ^99m^Tc‐GSA scintigraphy. The liver is a remarkable organ with regenerative capacity, enabling compensation for functional deficits [22]. Based on this mechanism, patients who are not candidates for hepatectomy due to limited liver volume initially have more opportunities for curative surgery through techniques such as PVE or associating liver partition and PV ligation for staged hepatectomy (ALPPS). However, each technique has its limitations. PVE is sometimes insufficiently effective, and tumor progression may occur during the prolonged waiting period (4–8 weeks) [3, 4]. In contrast, ALPPS achieves rapid liver hypertrophy within a short time; however, ALPPS has a high incidence of postoperative complications (53%–90%) and 90‐day mortality (0%–28.7%) [23]. It remains necessary to develop newer and more effective approaches to overcome these drawbacks. The combination of PVE and HVE has emerged as a novel strategy to improve both the efficacy and speed of liver hypertrophy while maintaining safety.

Nagino first described this technique, demonstrating its feasibility and promising results in 2003 [6]. Later, in 2009, Hwang successfully performed sequential ipsilateral HVE after PVE, achieving excellent and safe outcomes with a 27.6% ± 8.6% increase in FRLV [8]. HVE has been shown to be effective in cases of inadequate FLR hypertrophy following PVE, termed “salvage HVE” [7]. In 2016, Guiu introduced liver venous deprivation (LVD), which involved simultaneous trans‐hepatic portal and hepatic vein embolization [24]. Laurent referred to this approach as radiological simultaneous portohepatic vein embolization (RASPE) instead of LVD [9]. Le Roy later described a similar technique, termed biembolization (BE), which used a jugular vein approach for HVE and a transhepatic approach for PVE [10]. Guiu further advanced the technique by introducing extended LVD (eLVD), which combines embolization of the right PV, RHV, IRHV, and middle hepatic vein (MHV), demonstrating superior results in non‐cirrhotic patients [15]. Essentially, these techniques represent variations of combined PVE and HVE, differing mainly in the approach used and the timing of HVE relative to PVE.

Several studies have directly compared combined PVE–HVE with PVE alone, as summarized in Table 3. Numerous studies included a limited number of patients with various types of liver and biliary cancers, similar to our study, perhaps due to the stringent indication for embolization. The embolized hepatic vein in the combined PVE–HVE technique is almost exclusively the right hepatic vein, with or without MHV and/or IRHV. Most findings have demonstrated that combined PVE and HVE achieve a significantly greater FRL regeneration rate and KGR in terms of volume than PVE alone. Table 3 summarizes data from 13 studies, showing median values of the FRLV regeneration rate at 30.6% (27.0%–40.1%) in the PVE group and higher, at 52.6% (49.6%–60.6%), in the combined PVE–HVE group. Likewise, the combined PVE–HVE group demonstrates a higher KGR of FRLV at 18.1 (14.3–19.5) %/week compared to 9.8 (9.3–10.7) %/week in the PVE group. These results are consistent with our findings, as presented in Figure 3. In our study, HVE was performed proactively after PVE, with a median interval of 5 (3–7) days. The waiting time from PVE to surgery did not differ significantly between the PVE and sequential PVE–HVE groups. A potential advantage of simultaneous PVE–HVE over the sequential approach is the shorter waiting time from PVE to surgery and the requirement for only a single anesthesia procedure. However, simultaneous PVE–HVE can lead to excessive liver injury and even liver failure, especially in patients with impaired liver function. In contrast, the sequential approach allows time to assess the patient's response to PVE and provides the liver with a chance to recover before further intervention. A well‐designed prospective study should be conducted to clarify the comparative efficacy and safety of the two approaches.

**TABLE 3:** Overview of comparative studies on portal vein embolization versus portal and hepatic vein embolization.

First author^ref^, year	Patients, n	Type of combined PVE and HVE technique	Diagnosis	Embolized hepatic vein	FRLV regeneration rate, %	KGR, %/week or mL/day	Tool for assessing the changes of FRLF
	PVE/PVE–HVE		(Only PVE–HVE group)		PVE/PVE–HVE	PVE/PVE–HVE	
Hocquelet [25], 2018	6/6	Simultaneous	PHCC: 6	RHV: 6	31.3/67.0	—	—
Parano [26], 2019	16/13	Simultaneous	CRLM: 10; HCC: 3	RHV ± IRHV ± MHV: 13	—	4.8/16 (mL/day)	—
Guiu [27], 2020	22/29	Simultaneous	CRLM: 22; ICC: 4 HCC: 2; other: 1	RHV ± MHV: 29	18.6/52.6	—	^99m^Tc membrofenin HBS
Laurent [9], 2020	36/37	Simultaneous	CRLM: 23; ICC: 7 HCC: 4; others: 3	RHV: 37	29.0/61.2	—	—
Le Roy [10], 2020	41/31	Simultaneous	CRLM: 18; ICC: 2 HCC: 5; PHCC: 5; Other: 1	RHV: 27 RHV + MHV: 3 MHV: 1	31.9/51.2	8/19 (%/week)	—
Kobayashi [28], 2020	30/20	Simultaneous	CRLM: 10; HCC: 2 PHCC: 8	RHV: 18 RHV + MHV: 2	—	—	—
Masthoff [29], 2021	20/16	Simultaneous: 12 Sequential: 4	CRLM: 2; ICC: 4 HCC: 1; PHCC: 5 GC: 2; other: 2	RHV: 16	—	—	—
Heil [11], 2021	160/39 (7 centers)	Simultaneous	CRLM: 19; ICC: 4; HCC: 4; PHCC: 5; GC: 4; Other: 3	RHV: 27 RHV + MHV: 11 LHV + MHV: 1	48/59	13/21 (%/week)	—
Böning [13], 2022	14/14	Simultaneous	CRLM: 4; CC: 10	RHV: 14	44.9/48.2	—	—
Cassese [14], 2023	16/17	Simultaneous	CRLM: 17	RHV ± MHV: 17	27/49	4.8/10 (mL/day)	^99m^Tc membrofenin HBS
Araki [30], 2023	31/12	Sequential	CRLM: 1; ICC: 3 HCC: 2; PHCC: 6	RHV: 9 RHV + IRHV: 3	27/71.3	9.7/5.9 (%/week)	EOB‐MRI
Marino [12], 2023	19/12	Simultaneous	PHCC: 12	RHV: 12	42.8/52.5	9.9/17.1 (%/week)	^99m^Tc membrofenin HBS
Our study	24/8	Sequential	CRLM: 1; ICC: 2 HCC: 2; PHCC: 2 Other: 1	RHV: 6 RHV + IRHV: 2	26.2/35.5	7.4/13.1 (%/week) 4.4/7.2 (mL/day)	^99m^Tc GSA HBS
13 studies	435/254	Simultaneous: 230 Sequential: 24	CRLM: 127; ICC: 26 HCC: 25; PHCC: 59 GC: 6; other: 11	RHV ± IRHV ± MHV: 252 LHV + MHV: 1 MHV: 1	30.6 (27–40.1)/52.6 (49.6–60.6)^a^	9.8 (9.3–10.7)/18.1 (14.3–19.5) (%/week)^a^	

Abbreviations: ^99m^Tc‐GSA, technetium‐99m diethylenetriamine pentaacetic acid galactosyl human serum albumin; CC, cholangiocarcinoma; CRLM, colorectal liver metastases; EOB‐MRI, EOB‐magnetic resonance imaging; FRLF, future remnant liver function; FRLV, future remnant liver volume; GC, gallbladder cancer; HBS, hepatobilliary scintigraphy; HCC, hepatocellular carcinoma; HVE, hepatic vein embolization; ICC intrahepatic cholangiocarcinoma; IRHV, inferior right hepatic vein; KGR, kinetic growth rate; MHV, middle hepatic vein; PHCC, perihilar cholangiocarcinoma; PVE, portal vein embolization; RHV, right hepatic vein.

^a^ Median values with interquartile range of FRLV regeneration rates and kinetic growth rates were calculated based on available data across all studies mentioned above.

Most comparative studies have focused solely on changes in volume without evaluating the functional changes of the FRL, while what truly matters is the functional capacity of the FRL. In 2020, Guiu was the first to compare the FRLF regeneration rate following PVE versus combined PVE–HVE using ^99m^Tc‐mebrofenin hepatobiliary scintigraphy (HBS). At 3 weeks post‐embolization, the FRLF increased by 29.8% in the PVE group and 68.2% in the combined PVE–HVE group [27]. In 2023, Marino also utilized ^99m^Tc‐mebrofenin HBS to assess the functional hypertrophy of the FRL, reporting similar findings: 67.71% in the combined PVE–HVE group compared with 40.16% in the PVE group [12]. In 2023, Araki employed gadolinium ethoxybenzyl diethylenetriamine pentaacetic acid‐enhanced magnetic resonance imaging (EOB‐MRI) to evaluate post‐embolization changes in liver function. To our knowledge, no studies have utilized ^99m^Tc‐GSA scintigraphy to compare the change in FRLF after embolization between the two techniques, despite its value as an extremely useful tool for assessing the function of individual liver segments. ^99m^Tc‐GSA is a liver scintigraphy agent that binds to asialoglycoproteins (ASGPs), which are internalized into hepatocytes via a receptor that recognizes the nonreducing terminal galactose and N‐acetyl‐galactosamine residues of carbohydrate chains. These proteins are localized exclusively on liver parenchymal cells; therefore, the liver is the only uptake site for ^99m^Tc‐GSA, an ideal receptor‐targeted functional liver scintigraphy agent [31]. Other modalities, such as ^99m^Tc‐mebrofenin HBS and EOB‐MRI, also allow for segmental liver function evaluation; however, their accuracy may be compromised in patients with biliary obstruction, hyperbilirubinemia, or cirrhosis. In contrast, ^99m^Tc‐GSA scintigraphy is not influenced by serum bilirubin levels because bilirubin does not bind to asialoglycoprotein receptors. Moreover, ^99m^Tc‐GSA scintigraphy is also useful for assessing hepatic fibrosis, as the number of asialoglycoprotein receptors available for binding decreases in cirrhotic liver, reflecting impaired liver function. This tool is particularly useful for evaluating liver function [31]. The present study used the maximal removal rate of ^99m^Tc‐GSA (GSA‐Rmax) in the FRL obtained from ^99m^Tc‐GSA scintigraphy with a cutoff value of 0.15 mg/min per 50 kg of BW to predict postoperative liver failure and as a criterion for hepatectomy indication [16]. In our study, the FRL function evaluated by ^99m^Tc‐GSA scintigraphy increased markedly after intervention in both the PVE and sequential PVE–HVE groups (Figure 2C,D). This change was statistically significant only in the PVE group (*p* = 0.005), not in the sequential group (*p* = 0.086), likely due to the small sample size. However, the regeneration rate and KGR of FRLF are presented in Figure 3B,D, indicating 59.5% and 22.6%/week in the sequential PVE–HVE group, respectively, which are significantly higher than 25.2% and 8.6%/week in the PVE group.

The PVE procedure is a proven and widely used safe method [2], and studies on the combined PVE–HVE technique have also demonstrated its safety [11, 16]. In the present study, no cases of severe complications leading to death or requiring reintervention were observed in either the PVE group or the sequential PVE–HVE group. Although no significant differences in surgical outcomes were observed between the two groups, ISGLS grade B PHLF occurred in four patients (50%) in the sequential PVE–HVE group, as detailed in Table S3. These included cases involving either complex procedures (H15678‐B, H15678/2′3′) with prolonged operative time or impaired liver function. These cases were managed with minor treatments, including daily diuretics, albumin, and fresh‐frozen plasma, and showed early recovery. Notably, no patient in the sequential PVE–HVE group developed grade C PHLF.

The investigation of the mechanism underlying liver regeneration is critical for optimizing regenerative strategies. Marked liver regeneration occurs after partial hepatectomy (PH), and this process following PVE closely resembles that of PH. The portal venous flow to the non‐embolized liver lobe increases after PVE, as venous return from the splanchnic system remains largely unchanged, although this increase is slightly weaker than that observed after PH. This hemodynamic change affects the microstructure of the liver sinusoids and elevates shear stress, which is the functional force applied by blood flow on the endothelial surface. Consequently, a chain reaction is triggered, including the release of nitric oxide (NO), hepatocyte growth factor (HGF), vascular endothelial growth factor (VEGF), interleukin‐6 (IL‐6), and tumor necrosis factor‐α (TNF‐α), which are key factors in stimulating liver regeneration [5, 22].

In a rat model, expression of Ki‐67, a marker of liver regeneration, along with IL‐6, TNF‐α, and HGF, was greater and more prolonged in the portal and hepatic vein ligation group compared to the portal vein ligation (PVL) group [32]. In a pig model, simultaneous ligation of the portal and hepatic veins significantly reduced porto‐portal collaterals, which is recognized as a factor that diminishes hypertrophy compared to PVL alone [33]. However, the physiological processes underlying the effect of HVE have not yet been fully clarified and require further investigation through future studies in both clinical and animal models.

Recognizing the limitations associated with this study is essential. First, this was a retrospective study conducted at a single center, which may have introduced selection bias that affected the results. Second, the number of cases was relatively small and included a variety of underlying conditions. This was due to the strict criteria for HVE. Our findings need to be validated in large‐scale studies, potentially focusing on specific disease subgroups. Third, we did not assess the pathology of the two groups and the impact of liver fibrosis on hepatic hypertrophy. Further analysis is needed in this area to provide more specific indications for individual cases. In addition, a prospective study should be conducted to validate these findings and minimize inherent biases.

In conclusion, the present study demonstrates that sequential PVE–HVE is safe and practical, leading to a significant increase not only in the volume but also in the function of the FRL.

## Author Contributions

**Thanh Tung Lai:** data curation, formal analysis, investigation, methodology, visualization, writing – original draft, writing – review and editing. **Kosuke Matsui:** data curation, investigation, writing – review and editing. **Hideyuki Matsushima:** data curation, investigation, writing – review and editing. **Hidekazu Yamamoto:** data curation, investigation, writing – review and editing. **Gozo Kiguchi:** data curation, investigation, writing – review and editing. **Hisashi Kosaka:** data curation, investigation, writing – review and editing. **Van Khanh Nguyen:** data curation, investigation, writing – review and editing. **Yasuhiro Ueno:** data curation, investigation, writing – review and editing. **Shuji Kariya:** data curation, investigation, writing – review and editing. **Masaki Kaibori:** conceptualization, methodology, project administration, supervision, writing – review and editing.

## Ethics Statement

This study was approved by the institutional review board of Kansai Medical University (Approval number: 2024102). It was performed in accordance with the Declaration of Helsinki. This study received ethical approval for the use of an opt‐out methodology based on low risk to the participants.

## Conflicts of Interest

The authors declare no conflicts of interest.

## Supporting information


**Table S1:** Complications of embolization.
**Table S2:** Comparison of the changes in liver volume and function within each group before and after embolization.
**Table S3:** Clinical characteristics of patients with ISGLS grade B posthepatectomy liver failure in the sequential PVE–HVE group.
