# Supplementary material for: Efficacy of Sequential Hepatic Vein Embolization Following Portal Vein Embolization in Promoting Regeneration of Liver Volume and Function Before Right‐Sided Major Hepatectomy
Source: Ann Gastroenterol Surg. 2025 Aug 27;10(1):229–40. doi: 10.1002/ags3.70085 (PMC12757160; doi:10.1002/ags3.70085)
Supplement: Supplementary file 1 — Table S1: Complications of embolization. Table S2: Comparison of the changes in liver volume and function within each group before and after embolization. Table S3: Clinical characteristics of patients with ISGLS grade B posthepatectomy liver failure in the sequential PVE–HVE group. [file AGS3-10-229-s001.docx]

**Supporting information**

**Table S1. Complications of embolization**

| **Variable** | **PVE**  **(n=24)** | | **Sequential PVE-HVE**  **(n=8)** | | **P** |
| --- | --- | --- | --- | --- | --- |
| Migration of embolic material | 0 | (0%) | 0 | (0%) | - |
| Intra-abdominal hemorrhage, grade 1 | 2 | (8.3%) | 0 | 0%) | 1.000 |
| Fever, grade 1 | 0 | (0%) | 1 | 12.5% | 0.250 |
| Abdominal pain, grade 1 | 3 | (12.5%) | 1 | (12.5%) | 1.000 |
| Hepatic failure | 0 | (0%) | 0 | (0%) | - |
| Data are shown as n (%). Complications during and after embolization were classified according to CTCAE 5.0 | | | | | |

**Table S2. Comparison of the changes in liver volume and function within each group before and after embolization.**

| **Variable** | | **PVE (n=24)** | | | **Sequential PVE-HVE (n=8)** | | |
| --- | --- | --- | --- | --- | --- | --- | --- |
|  |  | **Before embolization** | **After embolization** | **P** | **Before embolization** | **After embolization** | **P** |
| **Liver volume** | | | | | | | |
|  | TLV, ml | 1135 (994 – 1355) | 1169 (978 – 1470) | 0.113 | 1308 (944 – 1503) | 1448 (1079 – 1538) | 0.036 |
|  | FRLV, ml | 411 (335 – 494) | 534 (424 – 593) | < 0.001 | 383 (352 – 458) | 521 (473 – 662) | 0.012 |
|  | FRLV/TLV, % | 34.6 (32.7 – 38.2) | 41.6 (36.3 – 49.0) | < 0.001 | 32.6 (25.4 – 37.8) | 43.0 (33.0 – 46.1) | 0.012 |
|  | FRLV/SLV, % | 37.5 (31.4-40.8) | 46.5 (40.5-52.3) | < 0.001 | 36.4 (31.3-39.5) | 48.5 (45.2-52.8) | 0.012 |
|  | FRLV/BW, % | 0.68 (0.57 – 0.88) | 0.90 (0.74 – 1.09) | < 0.001 | 0.69 (0.63 – 0.76) | 0.95 (0.92 – 0.98) | 0.012 |
| **Liver function – GSA Rmax (mg/min) by ^99m^Tc-GSA scintigraphy** | | | | | | | |
|  | Total liver function | 0.598 (0.421 – 0.774)^†^ | 0.528 (0.393 – 0.706)^†^ | 0.014 | 0.656 (0.607 – 0.884)^§^ | 0.626 (0.531 – 0.837)^§^ | 0.114 |
|  | Future remnant liver function | 0.294 (0.190 – 0.382)^†^ | 0.347 (0.231 – 0.453)^†^ | 0.005 | 0.249 (0.233 – 0.268)^§^ | 0.383 (0.352 – 0.472)^§^ | 0.068 |
| Data are shown as median (25th percentile to 75th percentile). SLV (ml) = 706.2 × BSA (m^2^) + 2.4. BSA (m^2^) = 0.007184 × (Weight in cm)^0.425^ × (Height in cm)^0.725^. PVE, portal vein embolization; HVE, hepatic vein embolization; FRLV, future remnant liver volume; TLV, total liver volume; SLV, standard liver volume; BSA, body surface area; BW, body weight; GSA Rmax – region maximal removal rate of ^99m^technetium-galactosyl human serum albumin; † (n=21), § (n=4). | | | | | | | |

**Table S3. Clinical characteristics of patients with ISGLS grade B posthepatectomy liver failure in the sequential PVE-HVE group.**

| No | Diagnosis | Surgical Procedure | FRLV/SLV before embolization (%) | FRLV/SLV  after embolization (%) | ALBI score | Blood loss  (ml) | Operative time (minutes) | Hospital stay (days) | Treatment |
| --- | --- | --- | --- | --- | --- | --- | --- | --- | --- |
| 1 | PHCC | H15678-B | 29.97 | 44.57 | -2.055 (grade 2b) | 1108 | 574 | 32 | Daily diuretics, albumin, FFP |
| 2 | PHCC | H15678-B | 31.98 | 49.24 | -2.615 (grade 1) | 2010 | 698 | 26 | Daily diuretics, albumin |
| 3 | CRLM | H15678/2’3’ | 36.57 | 47.70 | -2.161  (Grade 2b) | 933 | 383 | 27 | Daily diuretics, albumin |
| 4 | HCC | H5678 | 36.99 | 47.22 | -1.651  (Grade 2b) | 988 | 335 | 33 | Daily diuretics,  albumin, FFP |
| FRLV; furture remnant liver volume; SLV, standard liver volume; ALBI, albumin-bilirubin; PHCC, perihilar cholangiocarcinoma; CRLM; colorectal liver metastases; HCC, hepatocellular carcinoma; FFP, fresh-frozen plasma. Surgical procedures were classified arcoding to the “New World” terminology for hepatectomy. | | | | | | | | | |
